# BRAF Mutation Analysis in Primary Acral Melanoma of 41 Cases from South of Iran

**DOI:** 10.30699/IJP.20201.139458.2523

**Published:** 2021-07-06

**Authors:** Fatemeh Sari Aslani, Akbar Safaee, Mozhgan Akbarzadeh Jahromi, Leila Karami

**Affiliations:** 1 *Molecular Dermatology Research Center, Shiraz University of Medical Sciences, Shiraz, Iran *; 2 *Department of Medicine, Shiraz University of Medical Sciences, Shiraz, Iran*; 3 *Maternal-Fetal Medicine Research Center, Shiraz University of Medical Sciences, Shiraz, Iran*

**Keywords:** Acral Melanoma, BRAF, V600E, mutation

## Abstract

**Background & Objective:**

Acral melanoma (AM) is a common type of cutaneous melanoma that occurs in the skin of the palms, soles, and nail beds. This malignancy, like other types of cancer, has different genetic alterations. To date, despite decades of research the roles of oncogenic BRAF mutations in the pathogenesis of AM has not been fully clarified. The present study was designed to identify V600E mutation in patients with AM from the south of Iran.

**Methods:**

The samples were collected from the pathology lab archive of Shiraz University of Medical Sciences (2015-2020). A total of 41 patients with primary invasive AM underwent excisional biopsy or amputation were collected to evaluate BRAF V600E mutation using Polymerase Chain Reaction (PCR) and Sanger sequencing.

**Results:**

Total number of 41cases (21 male and 20 female) and age range of 34-87 years were enrolled. The histological subtypes were 24 acral lentiginous melanomas (ALM), 10 cases of nodular melanoma (NM), and 7 cases of superficial spreading melanoma (SSM). In our study, only one case (a 44-year-old male with nail bed AM and the histological subtype of acral lentigenous melanoma) showed BRAF-V600E mutation.

**Conclusion:**

These findings suggest that the population of our interest showed a very low prevalence of this mutation providing novel insights into the pathobiology of AM and its related treatment.

## Introduction

Acral melanoma is a subtype of skin cancer that occurs on acral skin, such as the palms, soles, and nails. Recent statistical analysis of some malignancies showed that AM is the most common cutaneous melanoma in some populations such as Asians and Africans (1). Histologically melanoma has been classi-fied into three major subtypes: Superficial Spreading Melanoma (SSM), Nodular Melanoma (NM) and Acral Lentiginous melanoma (ALM) (2). However, this classification particularly is population dependent. For instance, in populations with dark skin and Asian population, ALM is the most prevalent form of MM (3-5). ALM has also appeared to have a poorer prognosis among other subtypes of melanoma (6).

Despite decades of continuous studies, the mole-cular basis of this cancer is poorly understood (7). But it has been suggested that this type of melanoma similar to other types of melanomas, exhibits a high degree of genetic diversity. Mutations of melanoma have been extensively studied. The great group of mutations has already been evaluated in melanomas. For example, PIK3CA, PTEN, NRAS, KIT, and IDH1 have been studied; however, mutations of the BRAF have been paid lots of attention (8-10). 

BRAF is a cytoplasmic serine/threonine-protein kinase that orchestrates as a proto-oncogene in the mitogen-activated protein kinase (MAPK) pathway to regulate cell proliferation and survival (11). It has been commonly accepted that dysregulation of MAPK cascade plays a key role in tumorigenesis. 

Davies* et al. *for the first time showed that *BRAF* somatic missense mutations within the kinase domain (V600E) resulted in higher activity of BR-AF (12). This is the most common type of *BRAF* mutation and is located adjacent to an activating phosphorylation site at Ser599 (13). In addition, V600E mutated BRAF can bypass RAS function and activates a 500-fold increase of gene expression. To date, more than 40 BRAF mutations have been found and the majority of these mutations are located in the kinase domain and P-loop to increase MEK phosphorylation (14). Conversely, some mutations are reducing their activity. For instance, The D594V mutation of BRAF is called “kinase-dead”. Surprisingly, this mutation also exists in some cancers. Because this kinase-dead mutation has been shown to bind to CRAF to consequently more stimulating the MAPK-signaling pathway (15). Another mutation of the *BRAF* gene can produce recombinant AKAP9-BRAF oncogene formation. In this mutation, the BR-AF auto-inhibitory domains have been lost and finally, the MAPK pathway is constantly activated (16).

The study of mutations on *BRAF* is of interest because Vemurafenib as a potent inhibitor of mutated *BRAF* has great antitumor effects against melanoma with the *BRAF* V600E mutation but not against cells with other mutations (17). Developing studies have shown that this mutation is gently population depen-dent. In Chinese population, somatic mutations of the *BRAF* in melanomas have a high prevalence and approximately 22% of them have V600E mutations (18). Another study of the Australian population show-ed that approximately 33% of patients with melanomas have V600E mutation (19). In the American popula-tion, 34% of melanomas showed V600E mutation (20).

The present study was designed to identify V600E mutation in patients with AM from the south of Iran. To achieve this purpose human samples collected from the Dermatopathological Center of Shiraz University of Medical Sciences were subjected to mutation analysis based on Polymerase Chain Reaction (PCR) and Sanger sequencing for non V600E mutation.

## Material and Methods

Melanoma Tissue Samples

All samples were obtained from 41 patients (21 males and 20 female) of Dermatopathological Center of Shiraz University of Medical Sciences from 2015 to 2020. The selection of samples was performed based on the following criteria: the cases were histologically identified to be primary invasive AM, from excisional biopsy and amputation samples. All thin melanomas (<1mm) and the incisional biopsy specimens were excluded. Each diagnosis was verified by one expert dermatopathologist and an independent molecular path-ologist. The diagnosis & histologic features were revised according to CAP cancer protocol 2018. For better determination of PCR analysis, the positive control from known papillary thyroid carcinoma was used. The local ethical committee of the University of Shiraz University approved this study (Ethic number: IR.SUMS.MED.-REC.1339.68).

DNA Extraction

 The samples were cut in 5-7 μm thickness and collected in a 1.5 mL microcentrifuge tube. Consequently, DNA was extracted using an automated Nucleic acid extractor HP 16 PLUS kit (London, UK). The quality and purity of extracted DNA were evaluated by spectrophotometer Smart Spect Plus (Bio Rad, USA).

**ARMS-PCR and Sequencing**

The amplification refractory mutation system ARMS-PCR was performed as described previously. The primers consisted of 4 primers; forward outer: 5′-CTC TTC ATA ATG CTT GCT CTG ATA G-3′; reverse outer: 5′-GCC TCA ATT CTT ACC ATC CAC-3′; forward wild-type identifying: 5′-GTG ATT TTG GTC TAG CTA CAG T-3′ and reverse mutation identifying:5′-CCC ACT CCA TCG AGA TTT CT-3′. PCR examination was carried out in a 25 μL final volume containing 2.5 μL of lox reaction Buffer, 1.5-Mm OF 50 Mm mgcl2, 1 unit of Hotstar Taq DNA polymerase, 200 μM each dNTP, 400 nM primer Fo, 200 nM primer Ro, and Fiwt, 800 nM primer Rimut, and 50 ng genomic DNA template. PCR amplification process comprised denaturation at 95°C for 5 min, 40 cycles of 94°C for 20 s, 60°C for 20 s and 72°C for 20 s and final extension was performed at 72°C for 5 min. Products were then run on 2.5% agarose gel and visualized on the gel-documentation and analysis system (U:Genius 3, USA). Finally, dideoxy sequencing was performed by Sanger sequencing (ABI, USA).

Statistical Analysis

The SPSS version 21 was used for statistical analyses (SPSS Inc., Chicago, IL, USA). Quantitative data such as age was reported as the mean ± standard deviation, and qualitative data were presented in grouped data. The t-test was used to compare the age of patients, and the comparison of sexes between two groups was performed by chi-square test. In addition, logistic regression was used to compare BRAF V600E in groups. In all of this study P<0.05 was considered as the statistically significant difference.

## Results

Clinicopathological features

A total of 41 patients with AM were enrolled in the current study, including 21 males (51.3%) and 20 females (48.7%) with ratio of 1.05:1 and an age range of 34-87 years at the time of surgery. The most common tumor location was 33 (80.4%) in the foot (heel, plantar, and nail bed) and 8 (19.6%) in the hand (palm, finger, and nail bed). The pathological subtypes were as follows; 24 (58.5%) ALM, 10 (24.3%) were NM, and 7(17%) cases were SSM. Breslow tumor thickness ranged from 1.2 mm to 5 cm.

The Frequency of BRAF-V600E in Acral Melanoma

**Fig. 1:**
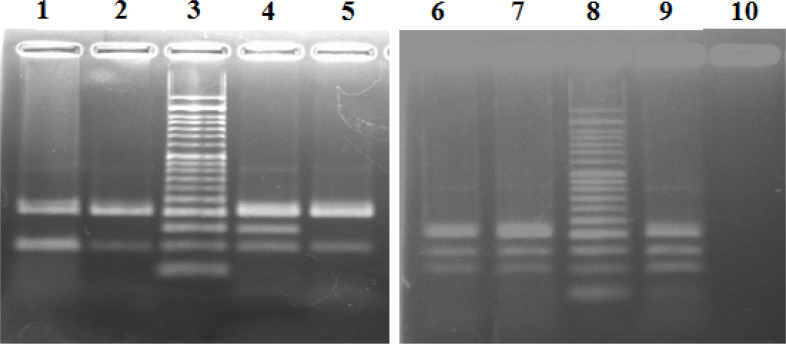
Analysis of PCR products from surgical samples of Acral Melanoma patients; all patients showed a 200 bp band. Patients of 1,2 and 5 showed only a 97 bp band exhibiting wild type alleles. Sample 7 shows 97 bp band and a sharp 144 bp band indicating the presence of wild type and mutant alleles (Heterozygous mutation). Sample 6 shows a 140 bp non- specific band and a 97 bp wild type band (this patient did not reveal V600E mutation in direct sequencing). Lane 4 and 9 are positive control (known papillary thyroid carcinoma) and lane 3 is 50 bp DNA ladder

**Fig. 2:**
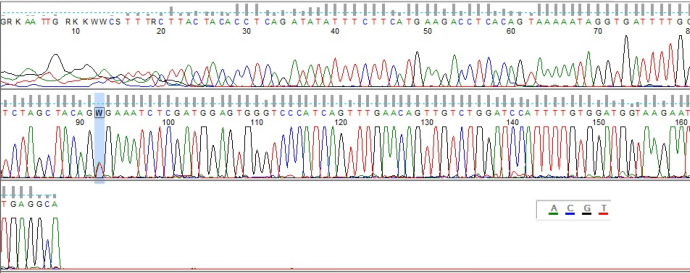
Direct Sanger sequencing of patient 7 revealed C.1799T>A (V600E) mutation

In the present study, we determined the frequency of the BRAF-V600E mutation in 41 specimens of AM. All samples were evaluated by PCR and Sanger sequencing. As indicated in Figure 1, PCR examination showed that among all 41 samples, the BRAF-V600E mutation was detected just in one sample (44 years old male patient with nail bed AM). The histological subtype of this case was acral lentigenous melanoma (ALM). Further analysis of Sanger sequencing was done on the negative and positive specimens (Figure 2).

## Discussion

It has been shown that more than 50% of melanomas harbor activating *BRAF* mutations. The most common type of this mutation is observed in a single nucleotide mutation (nucleotide 1799 T > A), resulting in the substitution of glutamic acid for valine. Another common mutation is substituting lysine instead of valine, which represents 5-6 % of mutations (21). This activating mutation also causes constitutive activation of some kinase and blocks the negative feedback mechanisms (22, 23).

There are few studies on BRAF mutation in Iranian patents with melanoma. For instance, Ghasemi* et al. *enrolled 82 cases of skin neoplasm from the north of Iran. They showed high intensity IHC staining expression of V600E in 60% of melanoma group but none of the other skin neoplasms and pigmented nevus showed high intensity staining (24). Despite the high prevalence of AM in Iranian population, no studies have been performed on the genetic pathway of this disease.

Here our data indicated that the population of southern Iran exhibited a low prevalence of BRAF-V600E mutation in AM which is the most prevalent subtype of MM. Our data showed that among all 41 samples of primary AM, just one specimen (2.43%) had a BRAF-V600E mutation without any nonV600E mutation. The histological subtype of this one was ALM. Our study also showed that BRAF mutation is not always necessary for tumorigenesis of AM. This prevalence is the lowest among related studies all over the world. In this line, Huang *et al*. reported that in the Taiwan population, approximately 10% of MM patients were positive for BRAF-V600E mutation (25). Ki Rang *et al*. showed *BRAF* mutation in 34.45% (22/64) of primary AM and ALM to be the most common histologic subtype (54.6%) followed by NM and SSM (7). Another similar study showed that regardless of the mutation prevalence, *BRAF*, and NRAS mutations are not necessary for melano-cytic tumor development (26). Moreover, using the mouse anti human *BRAF* monoclonal antibody and next generation sequencing on primary AM, Min Song Suh* et al. *showed that 46.3% of AM were positive for *BRAF* mutation. In parallel, it has been shown that VE1 immunohistochemistry analysis in close accordance with RT-PCR assay showed 32.2% positive *BRAF* mutation (4). However, Emi Dika *et al.* showed 12.9% *BRAF* mutation in of AM cases (27).

ALM was the commonest subtype in our study as other similar studies (3, 4). Hence, in some studies, NM has been more common (27). Also, the majority of previous studies evaluated different mutations, but unfortunately due to some limitations, this study was not able to assess another mutation suggesting that other types of mutations might be assessed in the future. With regard to our finding, and due to the sensitiveness of specific *BRAF* inhibitors, this study suggests that the prescription of such inhibitors to the population with a low prevalence of V600E has to be revised. However, much more study must be performed to confirm such results. Conversely, Lu *et al*. study of somatic mutations of MM showed that in Chinese population the *BRAF* gene mutation was 25.5% (110/432). They also showed that 89.1% of such mutations were V600E (23). In 2020 Takamichi detected *BRAF* V600E mutation in 18.8% of primary AM cases (21/112) by IHC staining of VE1 antibody. In that study tumor thickness was significantly associated with BRAF positivity (3). To emphasize the impact of this mutation, they also reported that patients with *BRAF* mutations are more likely to have ulceration in comparison to patients with-out *BRAF* mutations (28). The accumulating body of studies showed that *BRAF* V600E affects cancer cells in different mechanisms (29). 

This study was subjected to some limitations, including the inaccessibility of another mutation detection kit such as V600K, D594V, NRAS, and C-kit. Therefore, the authors recommend that such mutations’ detection can shed more light on this study. 

## Conclusion

In our study, as in other Asian population, ALM was the most common type of invasive AM, but unlike other studies due to low prevalence of *BRAF* mutation in our study the genetic disorder in our population seems to be something else, and more studies are needed for a more accurate conclusion.
